# Infant visual preference for the mother’s face and longitudinal associations with emotional reactivity in the first year of life

**DOI:** 10.1038/s41598-023-37448-8

**Published:** 2023-06-24

**Authors:** Silvia Rigato, Manuela Stets, Sophia Charalambous, Henrik Dvergsdal, Karla Holmboe

**Affiliations:** 1 Centre for Brain Science, Department of Psychology, University of Essex, Wivenhoe Park, Colchester, CO4 3SQ UK; 2 Business Administration Programme, Nord University, Bodø, Norway; 3 School of Psychological Science, University of Bristol, Bristol, UK

**Keywords:** Human behaviour, Social behaviour

## Abstract

Past research has focused on infants’ visual preference for the mother’s face, however it is still unknown how these responses change over time and what factors associate with such changes. A longitudinal study (N ~ 60) was conducted to investigate the trajectories of infant visual preference for the mother’s face and how these are related to the development of emotional reactivity in the first year of life. Two face stimuli (i.e., the infant’s mother and a consistent stranger face) were used in a visual preference task at 2 weeks, 4, 6, and 9 months of age. At each time point, mothers were asked to complete a measure of infant temperament via standardised questionnaires. Our results show that while at 2 weeks, 4 months and 9 months of age infants looked equally at both faces, infants at 6 months looked significantly longer at their mother’s face. We also observed prospective associations with emotional reactivity variables so that infants who looked longer at the mother’s face at 6 months showed higher falling reactivity, i.e. a better ability to recover from distress, at 9 months. We discuss these findings in light of the roles that both infant development and the caregiver play in emerging emotion regulation capacities during the first year of life.

## Introduction

From birth, infants show increased attention to conspecifics, mainly to their faces^1,2^, but also to their movements^3^, and their speech^4^. Furthermore, newborns prefer looking at faces that are socially more relevant to them, such as faces with direct gaze compared to faces with averted gaze^5^, happy faces compared to neutral facial expressions^6^, and also their mother’s face compared to a stranger’s face^7–9^. These initial biases are thought to be beneficial for the forthcoming experience with social compared to non-social information^10,11^.

Accordingly, infants’ ability to recognise caregivers’ faces (and voices) is critical for the development of early social interactions (e.g., attachment^12–14^). However, the literature on infant visual preference for mother’s face is mixed. Pascalis et al.^9^ found that newborns’ preference for their mother’s face disappeared when the outer contour was masked and only the inner features of the face were visible, suggesting that young infants use outer contour features to identify their mother. Another study showed that 6-month-old infants were able to discriminate between mothers’ and strangers’ faces only when they were dissimilar from each other; however, when the face of a stranger was similar to the infant’s own mother’s face, they showed no evidence of recognising their own mother^15^. In contrast to what was found with newborns^9^, this evidence suggests that at 6 months of age infants might instead use internal features for face recognition. Altogether, this might indicate that with age infants further develop their face recognition abilities by implementing a face processing mechanism for encoding second-order relational information^16^. The ability to distinguish the mother’s face from that of a stranger becomes in fact more robust with time between 3 and 10 months^17,18^, suggesting that progressively refined perceptual development allows infants to detect subtle features of their mother’s face^18,19^.

For typically developing infants, evidence for preference of mother’s face over that of a stranger are reported throughout the first half year of life (e.g., at birth^8^; at 1 month^20^; at 3.5 months^21^; at 5 months^22^; at 6-months^23^). However, there are also studies that report infant visual preference for faces of stranger, or no preference at all. For example, Melhuish^24^ found no evidence of recognition of the mother’s face in 1-month-old infants. Further, in a study with 3-month-old infants, Barrera and Maurer^25^ found that the initial preference for the mother’s face shifts to a stranger face when the mother’s face is presented repeatedly, suggesting that prolonged experience with visual stimuli, and the resulting habituation, influences infant preferences. Bartrip et al.^26^ also observed a shift from a preference for the mother’s face at 2 months of age to stranger’s faces at 5 months. While this could reflect effects of familiarity, so that a preference for familiar stimuli is present in the early stages of recognition, and a novelty preference appears only at later stages, it does not explain the controversial results from those studies that report preference for the mother’s face still present at 5 months or later (e.g.,^22,23^).

An important consideration is that such dissimilar findings in infant visual preferences might be related to individual differences, from cognitive abilities to motivation or temperament characteristics, as is also the case for general visual attention abilities. For example, it is well-established that infants who look for longer periods of time at stimuli process the information more slowly (e.g.,^27^), while faster learning is associated with short looks (e.g.,^28^). Short looking is generally regarded as an advantage for information processing (e.g.,^29^).

Evidence that individual differences play a key role in the processing of the mother’s face also comes from neuroimaging studies. An ERPs study with 6-month-old infants by Swingler et al.^32^ found that proximity-seeking behaviour during interactions with the mother was correlated with the processing of the mother’s face as reflected by the amplitude of the Negative central (Nc) component– a negative deflection occurring over fronto‐central electrode sites reflecting allocation of attentional processing resources^30,31^. A successive study from the same research group^33^ reported that 6-month-old infants’ distress and visual search for mother during separation correlated with face-related ERPs, and these associations were different for mother and stranger faces. Distress was specifically associated with processing of the mother’s face, while visual search for mother was associated with processing of the stranger’s face.

However, little is known about how temperament relates to behavioral visual preferences for mother and stranger faces in infancy. An eye-tracking study with infants in the second half of their first year of life reported evidence of associations between shyness and hypersensitivity to the eye regions when looking at faces of mother and strangers, however, it did not reveal differences in terms of visual preference towards these faces nor associations between these and temperamental characteristics^18^.

This body of evidence, together with studies that have revealed associations between early individual differences in visual attention and childhood temperament and behaviour development^34,35^, highlights the interactions between the factors that could potentially explain the mixed findings in looking behavior studies with familiar and novel faces. With the present work, we aimed to investigate the longitudinal trajectory of infant visual preference for the mother’s face from 2 weeks to 9 months of age and how changes in such preferences relate to individual temperament characteristics of emotional reactivity. At 4 time-points across the first 9 months of life, measures of infant temperament were collected through maternal report, and infant visual preferences towards the mother and a stranger’s face were assessed with a looking behavior task. In terms of trajectory of visual preference, existent literature shows that discriminatory abilities increase with age, therefore we expected infants at older ages to discriminate between the face stimuli by showing a preference for one of the two. However, while in general we could expect that an initial preference for the familiar face (mother) would shift to a preference for the novel stimulus (stranger) at later stages, the mixed findings available in the literature makes it difficult to predict a direction of such preference at the specific ages tested in this study. The analysis was therefore exploratory. Our second aim was to investigate how changes in looking behavior towards the mother’s face relates to infant temperament in the first year of life. Based on previous studies that have revealed significant associations between infant processing of the mother’s face and infant distress^33^, we specifically focused on infant emotional reactivity variables, i.e., negative affect, distress and falling reactivity. The term negative affect describes an individual’s state when they experience and express a negative emotion such as sadness, fear, or anger^36^. Infants high in distress are those who display fussing, crying, or startling behavior^37^. Falling reactivity, on the other hand, describes to what extent an individual is capable of recovering from a high level of distress or even positive excitement and, therefore, an infant’s ability to self-regulate^37^. Given the limited prior evidence linking infants’ looking time towards the mother’s face with their emotional reactivity, as well as the lack of studies systematically examining longitudinal associations, we did not have directional hypotheses with regard to the nature of potential relationships between these variables across time.

## Methods

### Participants

A total of 81 families were recruited from the University of Essex Babylab database and through events for expectant parents at Colchester General Hospital and other local venues in Essex, UK. Ethical approval was gained from the National Research Ethics Committee, London-Hampstead branch, United Kingdom (15/LO/0478). All methods were performed in accordance with the Declaration of Helsinki. Informed written consent was obtained from the parents of the infants prior to data collection.

The current manuscript reports on data from a longitudinal study investigating attention and social skills in the first year of life. While previously published articles include further details on the participant sample and other longitudinal data from these infants^38,39^, none includes data from the visual preference tasks reported in this manuscript.

Of the 81 recruited families, 16 mothers participated only in the prenatal assessment and could therefore not be included in the current analyses. Additionally, 2 infants suffered from complications at birth, and were excluded from the final sample. The sample reported on here consisted of about 60 infants (depending on data collection wave). The majority of participating infants were born full-term (at 37 weeks of gestation or later; *M* = 40 weeks, 3 days), and only two infants were born between 36 and 37 weeks of gestation. Infants in the sample had normal birth weight (*M*(*SD*) = 3.5(0.4) kg), no complications at birth, and no known health issues. The infants’ ethnic backgrounds were mainly White British (*N* = 54; 85.7%). Three infants were of Other White background (4.8%), four infants had mixed ethnic backgrounds but did not provide further details (6.4%), one infant was White British mixed with another ethnic background (1.6%), and one infant had a Hispanic background (1.6%).

Sixty-three infants came into the lab at 2 weeks of age and at 4 months, 61 infants came into the lab at 6 months, and 62 at 9 months of age. After excluding the datasets of those infants who did not complete the task (N = 11) and of those for which we experienced technical difficulties (N = 34) (for details see table S1 in Supplementary Material), the final sample consisted of 44 2-week-olds (17 females; M = 15.6 days, SD = 5.4), 58 4-month-olds (25 females; M = 124.8 days, SD = 8.6), 53 6-month-olds (23 females; M = 186.8 days, SD = 9.2), and 49 9-month-olds (19 females; M = 279 days, SD = 11.7).

### Stimuli

During a lab visit prior to childbirth, the expecting mothers had a picture taken of their face using a digital camera. The images showed fully frontal views of a mother’s head and neck with the face showing a friendly expression and a medium-large smile. Mothers were asked to look straight-ahead, directly into the camera. In order to prevent variation in the image material regarding lighting conditions and colorfulness, pictures were taken in front of the same white wall and under the same lighting conditions. Additionally, mothers were asked to remove earrings and to wear a white towel around their neck to ensure a high similarity in the neckline for all images. Subsequently, images were digitally processed using the Adobe Photoshop package, i.e., head-outlines were cut out from their background to eliminate potential shadows in the background and then placed onto a uniform, medium-grey background instead. When presented on the screen, faces had a width of 18.6 cm and a length of 21.2 cm (700 × 800 pixels, respectively) and the eyes of the two faces were presented level with each other and with the horizontal midline of the screen.

The stranger face selected for the visual preference task for each infant was a mother of another infant participating in the study (this image was kept constant at each visit). Selection criteria for finding an appropriate stranger were that the mothers and, consequently, the infants did not know the other mother and that they looked sufficiently different from each other without being extremely dissimilar. For instance, if a mother was wearing glasses when the image was taken, the respective stranger was selected such that she would be wearing glasses as well. Similarly, if a mother had her mouth closed when smiling, the respective stranger was selected such that she also had her mouth closed.

### Measures

#### Infant temperament

Mothers were asked to complete a well-established questionnaire relating to infant temperament, the Infant Behavior Questionnaire–Revised – Very Short Form (IBQ-R VSF;^40^) as well as two additional scales from the Infant Behavior Questionnaire–Revised (IBQ-R;^37^). The links to the online questionnaire were sent via email to those mothers who had confirmed attendance for a scheduled lab test session for the following day in an effort to keep the time difference between test session and questionnaire completion as short as practically possible.

The IBQ-R VSF consists of 37 items in total, and can be separated into three broad scales: Surgency (13 items; e.g., “When tossed around playfully, how often did the baby laugh?”), Negative Affect (12 items; e.g., “At the end of an exciting day, how often did your baby become tearful?”), and Orienting/Regulatory Capacity (12 items; e.g., “How often during the last week did the baby enjoy being read to?”)^40^. In addition to these scales, we also included the scales for Distress (12 items; e.g., “After sleeping, how often did the baby fuss or cry immediately?”) and Falling Reactivity (13 items; e.g., “When put down for a nap, how often did your baby settle down quickly?”) from the IBQ-R^37^. For each question, mothers were asked to rate how often their infant had shown a specific behavior in a given situation during the past week by selecting a score between 1 (“Never”) and 7 (“Always”). Additionally, an item could be scored as “NA – Does not apply” should the situation described in the question not have occurred over the previous seven days. Average scores were calculated for each scale. Items rated as “NA – Does not apply” were excluded from analysis.

For the purpose of the current study, which focused specifically on potential associations between visual preference and emotional reactivity, we only included the most relevant scales, i.e., Negative Affect, Distress, and Falling Reactivity.

Putnam et al.^40^ reported an average Cronbach’s alpha of 0.75 or above for the three broad IBQ-R VSF scales, and an average Cronbach’s alpha of 0.78 specifically for the Negative Affect scale. In the current study, Cronbach’s alpha for the Negative Affect scale ranged from 0.79 to 0.88 across ages, with a mean alpha of 0.82 (see Table S2 in Supplementary Material). Montirosso et al.^41^ reported average Cronbach’s alphas of 0.83 and 0.84 across two samples for the variables of Distress and Falling Reactivity, respectively. An overview of a number of studies using different versions of the IBQ-R revealed that Cronbach’s alpha for Distress and Falling Reactivity ranged between 0.74 and 0.79 (*M* = 0.76) and between 0.76 and 0.84 (*M* = 0.80), respectively, when using the short version of the IBQ-R^40^. In the current study, Cronbach’s alpha for the Distress scale ranged from 0.59 to 0.71 across ages, with a mean alpha of 0.65, and Falling Reactivity ranged from 0.65 to 0.85 across ages, with a mean alpha of 0.78 (see Table S2 in Supplementary Material). While the IBQ-R and IBQ-R VSF were developed and validated for infants aged 3–12 months^40^ (Putnam et al., 2014), research has indicated that these scales are also suitable for younger infants, including 2-week-olds^42,43^.

#### Visual preference task

**Figure 1:**
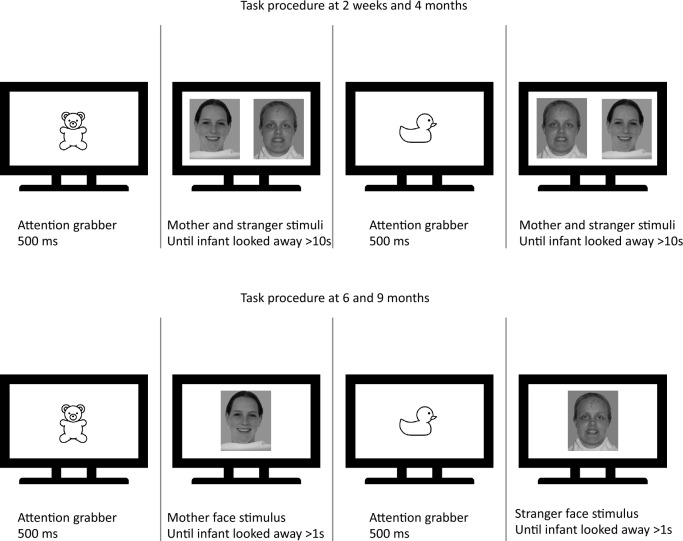
Visual preference task protocol used at 2 weeks and 4 months (top) and at 6 and 9 months (bottom). The image is for illustrative purposes only.

Eye‐tracking data were collected during a looking time paradigm to assess the infants’ visual preference for faces of mothers and strangers. Two visual preference paradigms are commonly used in the literature: simultaneous and serial presentation. In the first, two stimuli are presented simultaneously to the infant and measures of the infant’s gaze patterns during each trial are taken. In the serial paradigm, the infant is presented with a series of stimuli, one at the time, and fixation times to each are recorded and then compared. In order to keep the infant engaged throughout the task, and thereby maximise data collection at each time point, based on previous literature (e.g.^5,44,45^ for younger infants;^15^ for older infants, but see also^46^), we used the version of the paradigm that mostly fit the infants’ age (Fig. 1). Accordinlgy, the task used at the first and the second visit (at 2 weeks and at 4 months) consisted of a simultaneous stimuli presentation across at least 2 trials, or until the infant was attentive. Each trial began with a central attention getter, which was presented for 500 ms. The fixation stimulus was then followed by a pair of faces, the infant’s mother and a stranger’s face, which remained on the screen as long as the infants fixated one of them (i.e. infant control procedure). When they shifted their gaze from the display for more than 10 s, the experimenter moved to the next trial (see^5^). The location of the mother’s face was counterbalanced across trials. When infants returned to the lab for the 6- and 9-month visits, the task consisted of the same stimuli depicting mothers and strangers but presented individually, one after the other, in a serial paradigm. Each trial consisted of one mother and one stranger’s face, and this serial presentation was repeated 6 times. The order of presentation was counterbalanced across trials and randomized across participants. Each trial began when the infant first fixated at the first stimulus. The second stimulus was presented when the infant had been looking away from the first for at least one second (see^15^). If no fixation on the screen was detected for 2 s after stimulus onset, the presentation moved on to the next trial. Task presentation was controlled by custom-build software called Visual Tasks.

#### Eye tracking data acquisition and processing

The looking time data were recorded with a Tobii TX-300 eye‐tracking system (Tobii Technology, Stockholm, Sweden). The infant sat on their caregiver's lap at a 60 cm viewing distance from the 23″ eye‐tracker monitor. Once the experimenter had made sure that the infant's eye gaze was on the centre of the screen, testing began with a 5‐point calibration and 3‐point validation procedure during which an animated character moved across the screen following an unpredictable path to all four corners and into the centre of the screen. Data collection continued until all trials had been presented, or the infant had stopped attending to the presentation. The whole testing session was recorded with a high-speed camera at 300fps (ProMon Streaming High Speed Camera, Lake Image System, UK).

**Figure 2:**
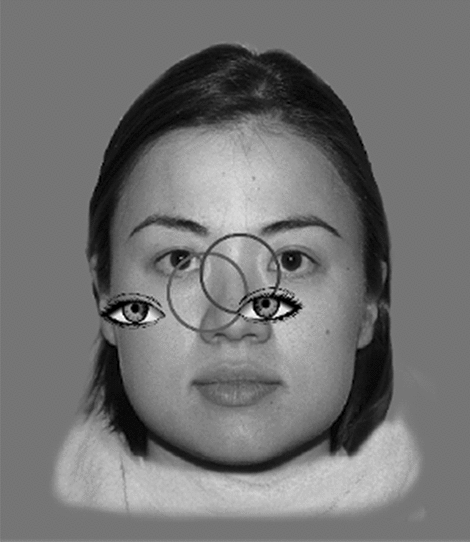
Example of how eye depictions and gaze positions (black and grey circles) were used to assess gaze data quality. The image is for illustrative purposes only.

Looking time data were coded by two independent coders using video recorded sources from both the eye tracking system and the high-speed camera. This combined method was employed to ensure a more accurate coding and inclusion of data samples which would have been otherwise lost by each of the recording systems separately (for a comparison between automatic eye tracking and manual coding, see^47^). Gaze data and video frames were synchronised with stimulus presentation via a small hidden area at the bottom corner of the screen that was lit up when stimuli appeared. The illumination state of this area was detected by a light sensor, transmitted to a Cedrus StimTracker (Cedrus Corporation, USA) and displayed on a small LED that was directly visible on the video recording of the infant’s face (recorded by the high-speed camera). The signal was also transmitted to the eye tracker and included in the gaze data. This setup ensured timing accuracy between stimulus onset, gaze data and video frames close to the eye tracker frame rate (3.3 ms). Without it, the accuracy would depend on the screen sync delay (5–25 ms) and various other unpredictable software and hardware delays. Our custom-built software used eye depictions to visualise the eye positions within the trackbox (see Fig. 2). The size of each eye depiction reflects distance from the screen. The positions were normalised to values between 0 and 1, corresponding to the boundaries of the trackbox and then transformed to screen coordinates and image sizes. Eye depictions were used to assess gaze data quality. The data was most reliable when both eyes were detected and located close to the centre of the trackbox. The black and grey circles in Fig. 2 show 2D gaze positions for the right and left eye respectively, projected on the monitor.

Coders coded the infants’ gaze location, e.g., fixating at the stimulus, looking away, as well as their more general behaviour, e.g. blinking, crying, and the respective timeframes. Coders were blind to the hypothesis of the study and had no knowledge of the infants’ temperament reports. Both coders coded 20% of the participants in each age group for the purposes of measuring intercoder reliability. Coding pairs achieved acceptable reliability (Cohen’s Kappa coefficient K) for the data in each age group (K _2 weeks_ = 0.70; K _4 months_ = 0.78; K _6 months_ = 0.70; K _9 months_ = 0.71).

## Results

After video coding the looking behavior, a few datasets were excluded from the analyses because the infants did not attend to at least one trial or did not look at each stimulus at least once within the same trial (two 2-week-olds, one 4-month-old). The final sample included in the analyses therefore consisted of 42 2-week-olds, 57 4-month-olds, 53 6-month-olds, and 49 9-month-olds. The average number of trials included in the analyses was 1.9 (SD = 0.5) for the 2-week-olds, 2.1 (SD = 0.6) for the 4-month-olds, 5.5 (SD = 1.1) for the 6-month-olds, and 5.7 (SD = 0.8) for the 9-month-olds.

### Looking time

**Figure 3:**
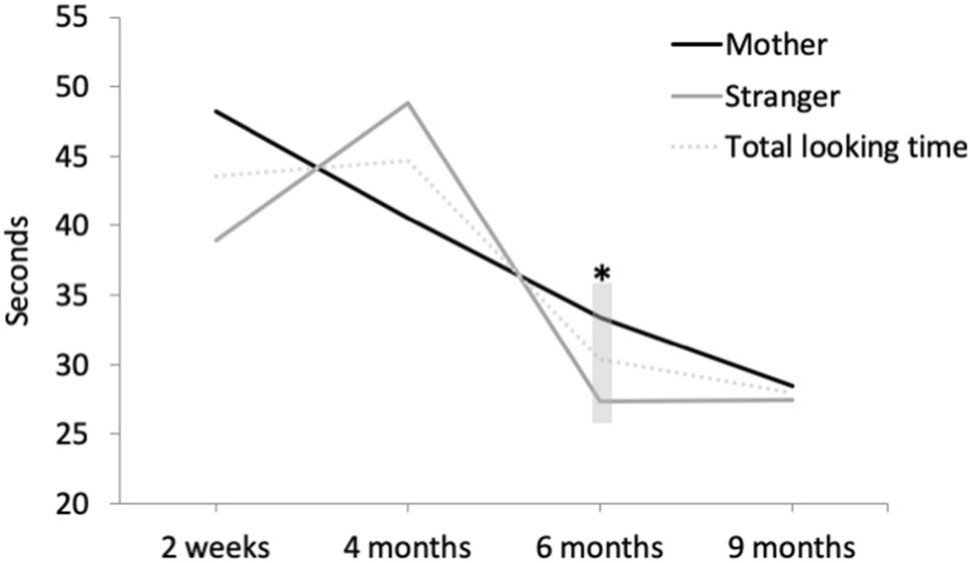
Trajectory of total looking time (in seconds) across stimuli (dotted line) and of looking time to mother (black line) and stranger face (grey line) stimuli at each age. A significant difference was found at 6 months.

To count for the age-related variation of the preferential looking task (different version from 6 months), two separate 2 × 2 repeated measures ANOVAs were run to test for Age and Face stimulus effects and their interaction at 2 weeks and 4 months, and 6 and 9 months, respectively. The analysis of looking time revealed no significant main effects or interactions at 2 weeks and 4 months, however there was a significant main effect of Face stimulus at 6 and 9 months, F(1,40) = 5.723, p = 0.022. This showed that at these ages infants preferred looking longer at their mother’s face (M = 30.6 s, SD = 12.6 s) than at a stranger’s face (M = 27.7 s, SD = 10.6 s), t(60) = 2.033, p = 0.04,* d* = 0.26. Because we were interested in identifying potential differences at individual age points we followed up with paired samples t-tests comparing looking time to mother’s and stranger’s face at each age. These revealed a significant difference at 6 months only, with longer looking times to mother’s face, t(52) = 2.759, p = 0.008,* d* = 0.40. Total looking times were not significantly different between mother and stranger faces at 2 weeks of age nor at 4 or 9 months of age, t(41) = 1.189, p = 0.241, t(56) = -1.581, p = 0.119, t(48) = 0.251, p = 0.803, respectively. Table 1 reports the mean and standard deviation (SD) of looking time (in ms) for each stimulus at each age. Figure 3 shows the trajectory of total looking time across stimuli as well as to each face stimulus across ages. An additional 4 × 2 repeated measures ANOVA where the looking time is converted into percentage and compared across the 4 age groups and 2 face stimuli can be found in the Supplementary material. This analyses revealed the same pattern of results as reported here.

**Table 1:** Mean and SD (in ms) of total looking time for mother and stranger face stimuli at each age.

Mean (SD)	2 weeks (N = 42)	4 months (N = 57)	6 months (N = 53)	9 months (N = 49)
Mother	48,202 (46,867)	40,544 (30,469)	33,392 (17,325)	28,637 (15,560)
Stranger	38,964 (31,773)	48,802 (31,039)	27,334 (12,508)	28,134 (13,157)

### Longitudinal correlations between looking time and emotional reactivity

Correlations were run to explore longitudinal associations between infant emotional reactivity and looking time to the mother’s face at each time point. For the purpose of these analyses, the percentage of looking towards the mother’s face was calculated for each infant by dividing the total sum duration of looking at the mother’s face by the total sum duration of looking at both the mother’s and the stranger’s face across trials. Table 2 shows all the correlations between looking time to mother’s face and emotion regulation variables (Negative Affect, Distress and Falling Reactivity) at each time point. While looking time to the mother’s face at 2 weeks was not associated with the temperament variables of interest at any age, we found positive correlations between looking time to mother’s face at 4 months and Falling Reactivity at 2 weeks of age, r = 0.284, p = 0.046, at 6 months, r = 0.297, p = 0.032, and at 9 months, r = 0.298, p = 0.032. Looking time to mother’s face at 4 months also correlated positively with Distress at 9 months of age, r = 0.323, p = 0.02. Looking time to mother’s face at 6 months was positively associated with Distress at 4 months, r = 0.324, p = 0.022, and with Falling Reactivity at 9 months of age, r = 0.454, p = 0.001. Only this latter correlation remained significant after controlling for multiple comparisons (Bonferroni correction, q = 0.001).

**Table 2:** Correlations between percentage of looking to the mother’s face and infant Negative Affect, Distress, and Falling Reactivity scale scores on the Infant Behavior Questionnaire – Revised, Very Short Form (IBQ-R VSF; Putnam et al., 2014) and Infant Behavior Questionnaire – Revised (IBQ-R; Gartstein & Rothbart, 2003) at each assessment stage.

		Looking time to mother’s face			
		2 weeks	4 months	6 months	9 months
2 weeks	Negative affect	0.156	− 0.039	0.197	− 0.099
	Distress	0.175	− 0.129	0.087	− 0.01
	Falling reactivity	− 0.064	0.284*	0.06	0.058
4 months	Negative affect	0.108	0.056	0.172	− 0.09
	Distress	0.155	0.112	0.324*	− 0.111
	Falling Reactivity	− 0.082	0.195	0.08	− 0.217
6 months	Negative affect	0.194	0.016	0.105	− 0.108
	Distress	0.218	− 0.016	0.078	− 0.053
	Falling reactivity	− 0.085	0.297*	0.168	− 0.205
9 months	Negative affect	0.306	0.134	− 0.102	0.087
	Distress	0.228	0.323*	− 0.047	0.02
	Falling reactivity	− 0.199	0.298*	0.454**	− 0.148

Significant correlations are in bold. **p < 0.01, *p < 0.05, two-tailed p-values.

## Discussion

The aim of the current work was twofold; we first sought to determine the longitudinal trajectory of visual preference towards the mother’s face in the first year of life, and then we investigated longitudinal associations between looking towards the mother’s face and individual infant emotional reactivity characteristics. Previous work suggests that infant discriminatory abilities for faces improve with age, and that while younger infants tend to show a preference for a familiar stimulus, there is then a shift to a preference for novel stimuli at older ages (e.g.,^26^). There is also a line of evidence indicating that processing of the mother’s face is related to distress in 6-month-old infants^33^. On this ground, we hypothesized that infants at older ages would show better discrimination between the mother’s face and a stranger’s face, and expected significant correlations between looking time to the mother’s face and emotional reactivity variables, i.e., negative affect, distress and falling reactivity. However, because little is known about the longitudinal relation between infant looking behavior towards the mother’s face and individual temperament characteristics, we did not have directional hypotheses with regard to the nature of potential associations between these variables across time.

In terms of longitudinal changes in visual preference towards the mother’s and a stranger’s face, we found a main effect of face stimulus only at 6 and 9 months, showing that at these ages infants prefer looking at their mother’s face. However, a reliable preference was only observed in infants at 6 months of age. At 2 weeks, 4 months and at 9 months, infants did not reliably show a preference for either of the face stimuli. These age-related differences may reflect developmental changes in how infants invest their attentional resources to familiar and novel stimuli, although caution is advised in interpreting the *overall change* in looking between 4 and 6 months as there was also a small change in experimental procedure between these ages.

The very few existent studies with 1-month-olds are not consistently reporting a solid visual preference at this young age^20,24^, therefore suggesting the possibility that infants in the first month of life are still learning about their primary caregiver’s features and may not reliably show a preference for them over other face stimuli. This interpretation fits well our results with 2-week-old infants who did not show a visual preference. On the other hand, we noticed that they generally spent more time looking at their mother’s face than a stranger’s face (though not significantly), which is in line with the existent evidence that under certain circumstances newborns can recognise their mother’s face^7–9^. It is however important to note that the experience as well as the neural maturation of a few-hour-old newborn, a 2-week-old and a 1-month-old infant largely differ (e.g.^48^), therefore it is difficult to directly compare their behavioural responses and to pinpoint the underlying mechanisms.

The absence of visual preference for the mother’s face at 4 months can be explained by the infants’ ability to quickly recognise her at this age; a 4-month-old infant might not need to spend long time and invest attentional resources looking at her mother’s face. Instead, it may be more adaptive for infants at this age to allocate attention to a novel face. In line with this view, although not statistically significant, in the present data we observe a rise of looking time towards the stranger face at 4 months (Table 1).

The preferential shift to the mother’s face at 6 months likely has a different underlying mechanism. In view of the present findings, as well as past research with infants of this age and younger (e.g.,^15,23^), it is quite unlikely that at 6 months infants are investing their attentional resources to still learn about and recognise their primary caregiver. Instead, we suggest that the time infants at this age spend looking towards their mother’s face reflect other aspects of developmental changes, including for example separation anxiety during a period that for many infants in the UK represents a transition between full time caregiving by their mother to a novel childcare environment, such as nursery. In line with this interpretation, Swingler et al.^33^ reported an association between processing of the mother’s face and infant distress during separation from the mother at 6 months of age.

In contrast, at 9 months, we expected that infants would have a solid recognition of their mother’s face and the absence of a reliable visual preference (despite the significant main effect in our overall analyses) might therefore indicate different cognitive processes, e.g., memory. Another possibility for the absence of preference at this age could be the employment of static face stimuli in our study, as infants rely on much more varied information to learn at this age, e.g. movement and language (e.g.^49^).

The second aim of the present study was to explore the associations between the looking time to the mother’s face and individual differences in emotional reactivity abilities in the first year of life. These correlations revealed that those infants who showed higher distress at 4 months of age, preferred to look at their mother’s face at 6 months. Although this correlation did not survive correction for multiple comparisons, it still shows some indication that early infant temperament may be associated with later looking behavior towards the mother’s face, and indeed is consistent with a neuroimaging study by Swingler et al.^33^ who found that distress was specifically associated with the processing of the mother’s face at 6 months of age. Additional positive associations were found between looking time to the mother’s face at 4 months of age and falling reactivity at all ages but not concurrrently. Although fragile as they did not survive correction for multiple comparisons, once replicated, these correlations could suggest that the amount of time a 4-month-old infant spends looking at her mother’s face might be an index of the infant’s ability to recover from a high level of distress or from a positive excitement.

More robustely, we found that looking longer at the mother’s face at 6 months was significantly associated with falling reactivity at 9 months of age. This might indicate that those infants who spend more time looking at their mother at 6 months develop better regulatory skills (i.e. management of reactivity) later on. One interpretation of these results altogether is that when infants are in distress, especially those who experience higher levels of negative arousal, might learn to orient towards their mothers – who would usually soothe them—as a regulatory mechanism that allows them to reduce distress (e.g.,^50^). In turn, looking at the caregiver, perhaps also as social referencing, will act as an adaptive behavior to gather information and further develop emotion regulatory skills. Indeed, the ability to self-regulate, which refers to processes that serve to modulate reactivity^51^, emerges from interactions with primary caregivers during the first years of life^52^. For example, Zarbatany and Lamb^53^ found that 14-month-old infants will selectively refer to their mothers (vs a stranger) to gather information about an ambiguous situation and adapt their behavior accordingly. The role of the caregiver is in fact paramount in facilitating emotion regulation, especially in the first six months of life when infants’ limited orienting and locomotor abilities urge them to rely on others to cope with novel or overwhelming situations. Ruff and Rothbart^54^ suggested that infants younger than 4 months show little control of orienting and the difficulty of disengaging from a visual stimulus force them to go through a period of “obligatory attention” that may lead to distress. However, by 4 months infants develop greater flexibility of orienting which has been found to be associated with lower parent-reported of negative emotionality and greater soothability in measures of infant temperament^55^. In line with the role of an orienting network that facilitates early emotional control (for a review see^51^), a study by Crockenberg and Leerkes^56^ found that by 6 months of age, infants are able to reduce their level of distress to novelty by looking away from the novel stimulus, re-orient towards their mother and then engage in something else, even when mothers are not actively engaged. The authors conclude that either merely seeing their mothers exercises a regulatory effect or that by 6 months, infants have learned to modulate arousal by redirecting their attention. While further investigation is needed to fully elucidate these mechanisms, such findings suggest that both infant social attention development and the caregiver play a crucial role in the development of emotion regulation in the first year of life. Here, we specifically found that a visual preference for the mother’s face at 6 months might be adaptive and facilitate the development of the infant’s ability to recover from a high level of distress or excitement and therefore to self-regulate at 9 months.

In summary, the present study suggests that the mixed findings in the existing literature on infant visual preference for the mother’s face might reflect age-related developmental changes as well as associations with individual temperament characteristics that develop with time. While this is one of the few longitudinal studies that systematically investigated infant looking behavior towards the mother’s face and its association with emotional reactivity across time, there are some limitations that need to be considered in interpreting the results. First, we employed two slightly different versions of the looking task according to the infants’ age. While this was done to maximise the chances of successful longitudinal data collection, it also makes a direct comparison of looking times across the first two (2 weeks and 4 months) and last two (6 and 9 months) ages more difficult. Second, we obtained data on infant temperament from a single informant, i.e., the mother, while it would be ideal to collect data from multiple informants and ideally include direct observations of infant behavior. It would also be informative to add measures of mother-infant attachment to investigate whether their developing relationship across the first year of life is associated with the infant’s preference for the mother’s face. Finally, we experienced a relative large amount of data loss (about 20%) due to technical error in the 2 weeks and 9 months age group. This impacted on the sample size of our study whose findings would be strengthened if replicated in a larger sample in the future. Despite these limitations, the current study provides novel insights into the development of visual preferences for the mother’s face versus a stranger’s face and the unfolding associations between looking behavior towards the mother’s face and individual differences in emotional reactivity skills across the first year of life.

## Supplementary Information

Supplementary Information.

## Data Availability

Anonymized individual questionnaire scale scores and visual preference looking times are publicly available on https://osf.io/9yrfh/?view_only=9f43662b935a4b81b86441fae35017b9.
