# Supplementary material for: Infant visual preference for the mother’s face and longitudinal associations with emotional reactivity in the first year of life
Source: Sci Rep. 2023 Jun 24;13:10263. doi: 10.1038/s41598-023-37448-8 (PMC10290679; doi:10.1038/s41598-023-37448-8)
Supplement: Supplementary file 1 — Supplementary Information. [file 41598_2023_37448_MOESM1_ESM.docx]

**SUPPLEMENTARY MATERIAL**

**Infant visual preference for the mother’s face and longitudinal associations with emotional reactivity in the first year of life**

Silvia Rigato^1^, Manuela Stets^1^, Sophia Charalambous^1^, Henrik Dvergsdal^2^,

and Karla Holmboe^3^

^1^Centre for Brain Science, Department of Psychology, University of Essex

^2^Business Administration Programme, Nord University

^3^School of Psychological Science, University of Bristol

*Participants*

Table S1 reports information on the number of participants who were invited to take part in the study, those who came into the lab, reasons for excluding data from analyses, and final sample at each age of assessment.

|  | *Invited to lab* | *Came in lab* | *Excluded* | | | *Final sample* |
| --- | --- | --- | --- | --- | --- | --- |
|  |  |  | *Technical error* | *Fussiness* | *Not meeting criteria for looking time* |  |
| *2 weeks* | 73 | 63 | 13 | 6 | 2 | 42 |
| *4 months* | 70 | 63 | 4 | 1 | 1 | 57 |
| *6 months* | 67 | 61 | 5 | 3 | - | 53 |
| *9 months* | 67 | 62 | 12 | 1 | - | 49 |

**Table S1.** Overview of number of participants invited to the lab, those who took part in the study and those excluded from analyses.

*Scale reliability*

Table S2 reports Cronbach’s alpha for the scales Negative Affect, Distress, and Falling Reactivity at each assessment stage. Please note that the low Cronbach’s alpha for the scales of Distress and Falling Reactivity at 2 weeks of age is due to some of the items not being applicable at that young age (e.g. “After sleeping, how often did the baby play quietly in the crib?”; “When your baby was upset about something, how often did s/he soothe

her/himself with other things (such as a stuffed animal, or blanket)?”).

|  | *Negative Affect* | *Distress* | *Falling Reactivity* |
| --- | --- | --- | --- |
| *2 weeks* | .878 | .585 | .653 |
| *4 months* | .791 | .664 | .845 |
| *6 months* | .815 | .705 | .807 |
| *9 months* | .803 | .640 | .829 |

**Table S2.** Cronbach’s alpha for Negative Affect, Distress, and Falling Reactivity scale scores on the Infant Behavior Questionnaire – Revised, Very Short Form (IBQ-R VSF; Putnam et al., 2014) and Infant Behavior Questionnaire – Revised (IBQ-R; Gartstein & Rothbart, 2003) at each assessment stage.

*Additional analyses*

*Looking time*

An additional 4x2 repeated measures ANOVA where the looking time is converted into percentage and compared across the 4 age groups and 2 face stimuli was conducted. The percentage of looking towards the mother’s face was calculated for each infant by dividing the total sum duration of looking at the mother’s face by the total sum duration of looking at both the mother’s and the stranger’s face. The analysis revealed a significant interaction between Age and Face stimulus, F(3,72) = 5.098, p = .008. Because we were interested in identifying potential differences at individual age points we followed up with paired samples t-tests comparing looking time to mother’s and stranger’s face at each age. These revealed a significant difference at 6 months only, with longer looking times to mother’s face, t(52)=2.751, p=.008, *d*=.76.

**References**

Gartstein, M. A., & Rothbart, M. K. (2003). Studying infant temperament via the Revised Infant Behavior Questionnaire. *Infant Behavior and Development, 26(1)*, 64-86.

Putnam, S. P., Helbig, A. L., Gartstein, M. A., Rothbart, M. K., & Leerkes, E. (2014). Development and Assessment of Short and very Short Forms of the Infant Behavior Questionnaire-Revised. *Journal of Personality Assessment*, 1-14.
